# A multi-index approach to assessing foraging mode: a case study using chameleons

**DOI:** 10.1093/cz/zoaf065

**Published:** 2025-09-17

**Authors:** Wade K Stanton-Jones, Krystal A Tolley, Jody M Barends, Graham J Alexander

**Affiliations:** South African National Biodiversity Institute, Kirstenbosch Research Centre, Private Bag X7, Claremont, Cape Town 7735, South Africa; School of Animal, Plant and Environmental Sciences, University of the Witwatersrand, P.O. Wits 2050, Johannesburg, South Africa; South African National Biodiversity Institute, Kirstenbosch Research Centre, Private Bag X7, Claremont, Cape Town 7735, South Africa; Department of Zoology, University of Johannesburg, P.O. Box 524, Johannesburg 2006, South Africa; South African National Biodiversity Institute, Kirstenbosch Research Centre, Private Bag X7, Claremont, Cape Town 7735, South Africa; Department of Zoology, University of Johannesburg, P.O. Box 524, Johannesburg 2006, South Africa; School of Animal, Plant and Environmental Sciences, University of the Witwatersrand, P.O. Wits 2050, Johannesburg, South Africa

**Keywords:** Africa, *Bradypodion*, chameleons, foraging mode, radio-telemetry, reptiles

## Abstract

Classifying the foraging mode of a species is challenging and is usually assessed through movement-based indices. Without considering what motivates movement, these indices may result in a biased interpretation of foraging mode—overlooking the fact that movement is influenced by multiple biological factors and not solely foraging. Conversely, while attack-based indices provide a more informative assessment of foraging mode because behavior is incorporated, the most comprehensive and reliable assessments should incorporate both movement- and attack-based indices. We applied a combination of movement- and attack-based indices to reevaluate foraging mode in chameleons, using *Bradypodion pumilum* as a case study. Focal observations were used to record the behaviors of 38 individuals, 12 of which were telemetered over two 10-day periods. Chameleons spent most of the day in stationary positions, perched in direct sunlight during mornings but in partial sunlight or shaded areas during afternoons. From >100 feeding events, chameleons had a significantly higher percentage of attacks and rate of attacks on prey while stationary than when moving. Most recorded intraspecific interactions were between females and males. Not only do our findings suggest that chameleons are motivated to move for several reasons but after considering life history traits in conjunction with our findings, we propose a paradigm shift in chameleon foraging mode suggesting that they should be considered ambush foragers instead of “cruise foragers” as previously classified. Overall, our findings demonstrate the importance of a multifaceted approach to foraging mode assessments, and we caution against using only one type of index in evaluations.

## Teaser

This study provides a comprehensive assessment of foraging mode, using chameleons as a case study. Herein, a combination of movement- and attack-based indices, motivators of movement, and consideration of life history traits, are used to classify foraging behavior.

## Graphical Abstract

**Graphical Abstract:**
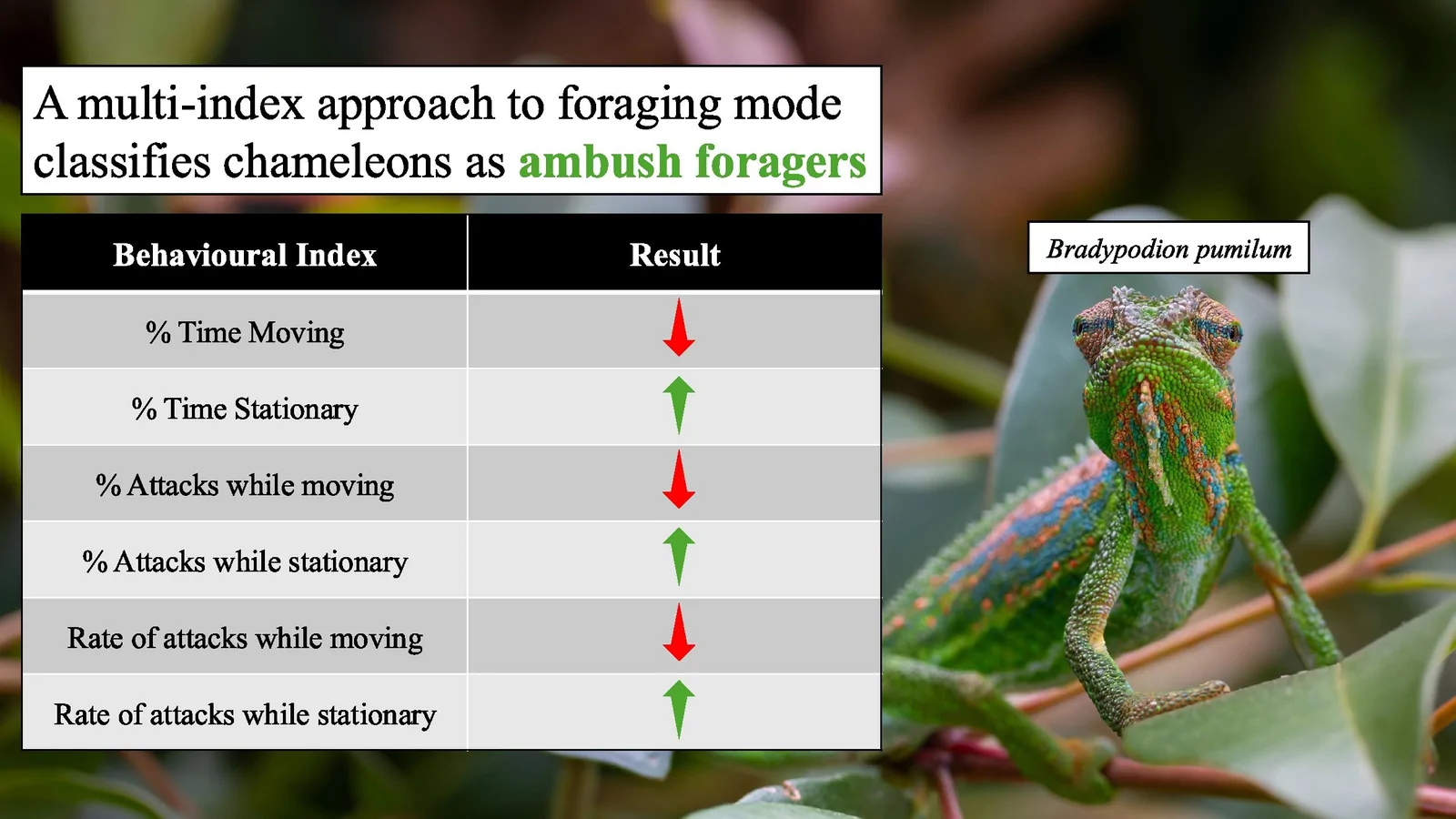

The generally accepted paradigm for foraging strategy is to classify predators within a dichotomy—those that sit-and-wait for prey to come to them (sit-and-wait or ambush foragers), and those that actively search for prey items (active foragers) (Pianka 1966; Schoener 1971; Huey and Pianka 1981). However, the new paradigm of lizard foraging suggests that their foraging strategies are on a continuum with the accepted dichotomy representing the extremes (McBrayer et al. 2007). Overall, there has been a profusion of studies of squamate reptiles that suggest that the characteristics of foraging mode for some species are complex and can be influenced by several factors, including life stage (Shine and Wall 2007), habitat use (Johnson et al. 2008), seasonality (Nemes 2002), and prey type (Glaudas et al. 2019). Thus, it is becoming increasingly more apparent that correctly classifying foraging mode can be challenging.

Early assessments (e.g., Huey and Pianka 1981) on classifying foraging mode were relatively simple, focusing on movement-based indices such as moves per minute (MPM) and percentage time spent moving (%TM) as the basis of assessments. Many subsequent studies also used these metrics to assess foraging behavior, particularly in lizards (e.g., Cooper et al. 1997; Du Toit et al. 2002; Mori and Randriamahazo 2002; Butler 2005; Hagey et al. 2010). While these movement metrics provide some insight into aspects of the foraging modes of lizards, they are also impacted by behaviors that are not related to foraging. For example, lizard movements may also be related to mate searching (Kerr and Bull 2006 ), mate attraction (Leu et al. 2016), homeostatic regulation (Stanton-Jones et al. 2018; Dezetter et al. 2023), predation avoidance (Cooper et al. 2010), and ambush site selection (Anderson 2007). These factors are all motivators of movement in reptiles. Thus, to avoid error in foraging mode classification, it is important to consider the motivation behind the movement (i.e., an assessment of patterns across different behaviors) when making assumptions about a species foraging behavior (Alexander and Wang 2023).

Some studies have recognized the shortcomings of relying solely on movement-based indices to assess foraging mode and proposed that variables directly relating to foraging behavior also be used in assessments. These variables are exclusively associated with feeding events whereby attacks made on prey are recorded according to the feeding animal's behavior while making the predatory attack (i.e., while moving or stationary). These variables have been referred to as “attack-based” indices. For example, Cooper et al. (1999) proposed that percentage of attacks made on prey while moving (PAM) be included in assessments, while Williams and McBrayer (2011) suggested the inclusion of the percentage of attacks made on prey while stationary (PAS). Another important attack-based index that is not often recorded is the rate of attacks on prey, which provides a measure of feeding events per unit time (e.g., Williams and McBrayer 2011). Although a significant correlation between PAM and %TM has been found for some species, PAM and MPM do not appear to be correlated (Cooper et al. 1999). However, both %TM and MPM provide some information on a species foraging behavior but should be integrated with attack-based indices. The collective results will likely offer a more accurate assessment of foraging mode. However, PAM and PAS require data on feeding events, which can be a time-consuming process to collect, especially for infrequently feeding animals like most reptiles. Presumably, this is why there are few studies that incorporate attack-based indices into assessments of reptile foraging modes and why most studies have exclusively used movement-based indices in assessments on foraging mode.

Investigations into chameleon foraging modes are few and, to our knowledge, are limited to only two published studies (Butler 2005; Hagey et al. 2010). Both studies posit that chameleons are neither ambush nor active foragers and instead classified them as “cruise foragers” (i.e., move in search of prey, stop and scan for prey, and move again; Regal 1983). However, these studies focused on movement-based indices and paid little attention to feeding events in their analyses. Thus, the conclusion that chameleons are cruise foragers was established upon the assumption that chameleons are motivated to move mainly to forage. In addition, neither study included thermoregulatory behavior as an independent factor, nor considered the possibility of intraspecific interactions as a motivation to move. As with other ectothermic animals, chameleon body temperature (T_b_) is greatly influenced by environmental temperature of the animal's microhabitat and thus regulated by behavioral adjustments (Bennett 2004). Behavioral thermoregulation is commonly used by reptiles so that individuals can achieve a selected or target T_b_ to facilitate optimal performance (Seebacher and Franklin 2005). Even though chameleons should be able to forage effectively over a broad thermal range (Anderson and Deban 2010; Segall et al. 2013), raising their T_b_ is still important for other physiological traits (e.g., digestion, movement, reproduction, etc.). This suggests that foraging may not be the only motivator for movement in chameleons, but that movement is motivated by a combination of physiological and ecological factors.

*Bradypodion pumilum* (Cape Dwarf Chameleon; Gmelin 1789) is an arboreal dwarf chameleon, endemic to southwestern South Africa (Tolley and Burger 2007). There are currently two recognized ecomorphs of this species; one inhabiting fynbos vegetation (hereafter, fynbos ecomorph), and the other inhabiting woodland/forest habitats (hereafter, forest ecomorph) (Tolley and Burger 2007; Hopkins and Tolley 2011; Tolley et al. 2019; Barends et al. 2025). These ecomorphs appear to have different prey preferences with the forest ecomorph consuming mainly active prey while the fynbos ecomorph tends to forage on both sedentary and active prey (Measey et al. 2011). Butler (2005) investigated the foraging behavior in the *B. pumilum* forest ecomorph and challenged the conventional dichotomy of foraging modes by arguing that their foraging behavior is similar to that of an active forager, despite MPM being relatively low. Thus, *B. pumilum* was classified as a cruise forager (Butler 2005). Not only did the assessment place little priority on feeding events, but it did not consider the motivation for movement behaviors unrelated to foraging such as mate-searching, mate-guarding (see Rebelo et al. 2022), other interactions with conspecifics (e.g., sexual displays) or thermoregulation. Thus, further investigations of the movements of this species, the behavior of individuals when feeding, and life history characteristics presents an opportunity to reevaluate foraging mode in chameleons, and to assess whether the cruise foraging hypothesis is supported, or if one of the conventional modes of foraging is more appropriate.

To evaluate the efficacy of approaches to accurately classify foraging mode, we compared the outcomes movement and attack-based methods using the same population of *B. pumilum* studied by Butler (2005). We recorded movement and stationary behaviors of chameleons (i.e., %TM and the inverse, percentage time spent stationary, %TS), feeding events (i.e., PAS, PAM, attack rates), and movement not specifically related to foraging (i.e., moving into different thermal microhabitats, and movement related to intraspecific interactions). We also interpreted our findings in light of the species life history characteristics (e.g., morphology, physiology, and behavior). This more comprehensive approach allowed us to reevaluate foraging mode in *B. pumilum*. We predicted that our refined methodological approach that incorporates a full suite of behaviors might not support the notion that chameleons are cruise foragers and instead may provide a new paradigm on their foraging mode.

## Materials and methods

### Study site

**Figure 1:**
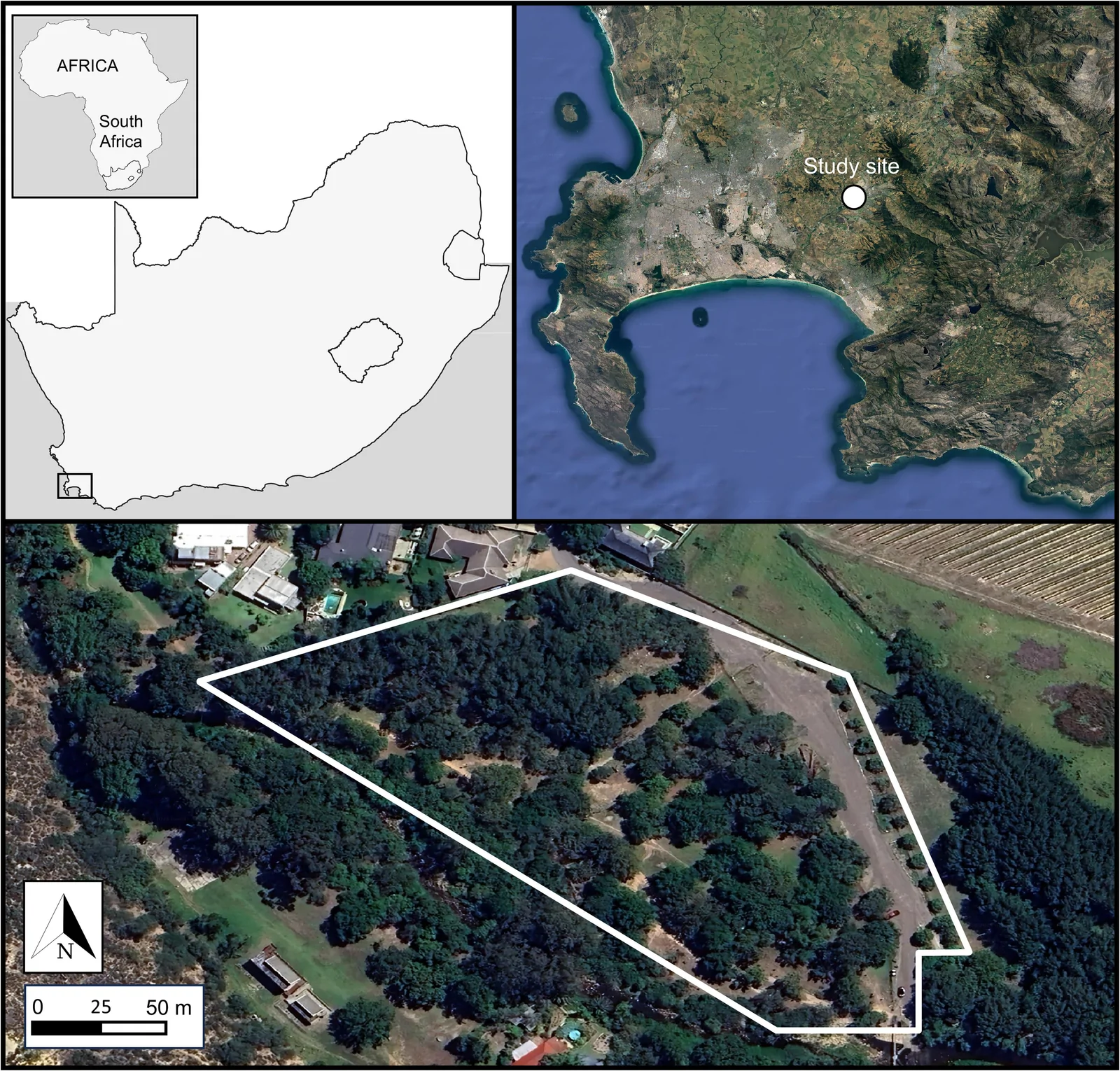
Overview of the study site within which individuals of *Bradypodion pumilum* were radio-tracked and behaviors observed at Brümmer Park, Stellenbosch, Western Cape, South Africa (white border). The maps of South Africa (including Eswatini and Lesotho), Stellenbosch and surrounding areas in the Western Cape Province, and Brümmer Park were created using QGIS Version 3.36 (www.qgis.org) with Google satellite imagery (www.google.com/maps).

We observed and recorded the behaviors using focal animal observations of 38 individuals of *Bradypodion pumilum* (Cape Dwarf Chameleon) for 10 days in April 2024 and 10 days in February 2025 from a population in the town of Stellenbosch, Western Cape Province, South Africa. Specifically, the population occurs in Brümmer Park (33° 56′ 25.3″S; 18° 53′ 23.0″E, 140 m.a.s.l.), a recreational park adjacent to the Eerste River, characterized by a mixture of indigenous and exotic vegetation (Fig. 1). The riparian forest habitat offers a cool thermal niche that is comprised of a mixture of native and exotic species composed of large trees that are sometimes interconnected at the canopy. There is a variety of low shrubs and small trees, but the park lacks continuous ground cover, which has been replaced by lawn and pathways.

### Chameleon capture and radio-tagging

Chameleons were located at night using torchlight when they visually stand out against the vegetation. The coordinates of each individual were recorded (+/−3 m precision) using a Garmin GPSMAP® 64 GPS. Sex was recorded, along with body mass and snout-vent lengths (SVL) to the nearest 0.1 g and 0.1 mm, using a digital balance and calipers, respectively. All chameleons were marked with a unique identification number (ID) on the posterolateral surfaces of their bodies, directly anterior to the hind limbs, using a non-toxic surgical skin marker (Tondaus®). The purpose of documenting the identity of all encountered individuals was to ensure that intraspecific interactions with the telemetered individuals could be recorded.

**Figure 2:**
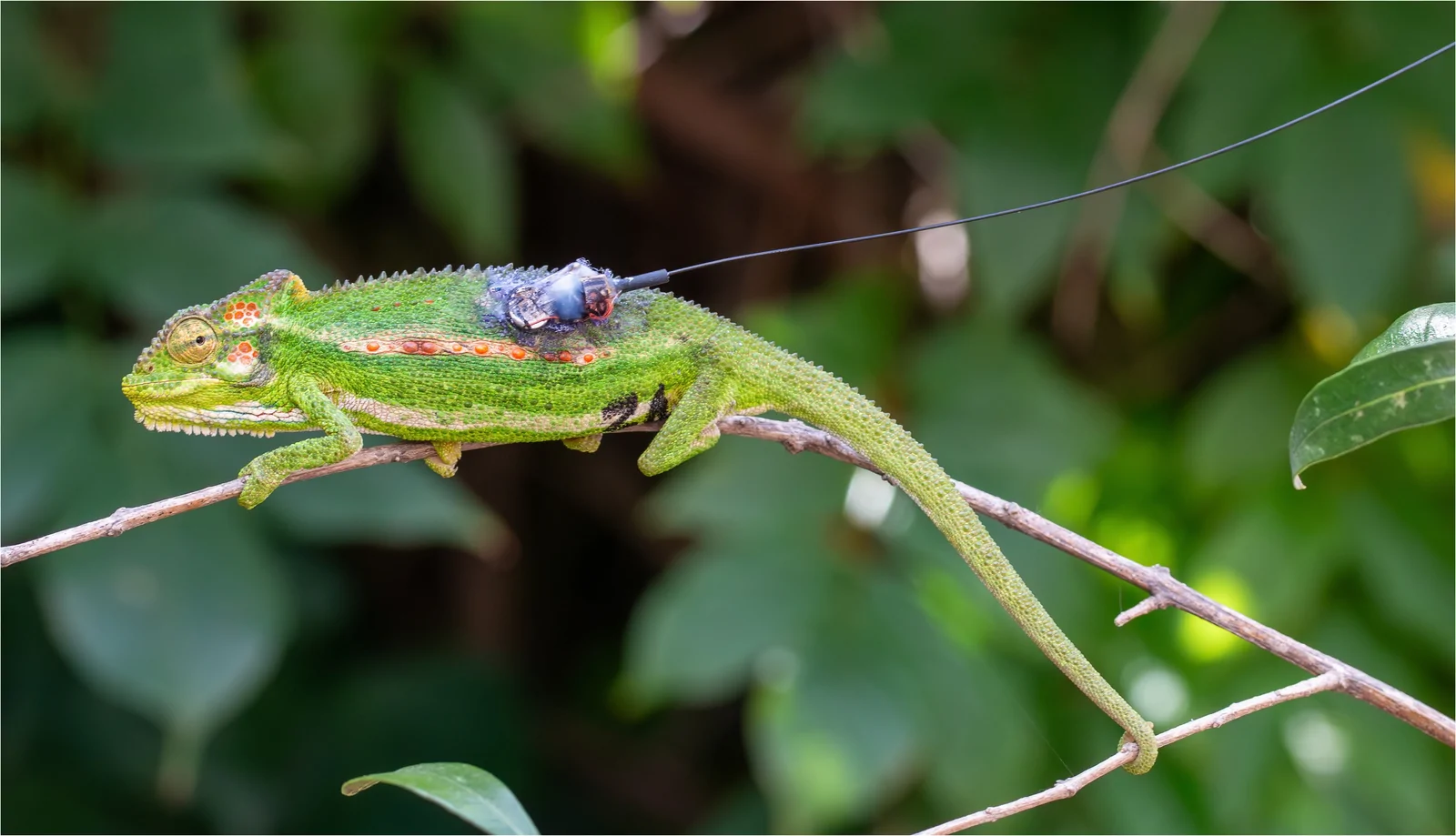
An adult female *Bradypodion pumilum* with a Holohil radio-transmitter (LB-2X) secured to the midbody flank using Histoacryl^®^ tissue glue. Photograph by W.K.S.-J.

Twelve adult individuals (six per field season) of at least 7 g in body mass (three females and three males) were temporarily removed from the site and kept overnight for fitment of a radio-transmitter the next day. Following the methodology of Rebelo et al. (2022), a 0.31 g radio-transmitter (Holohil LB-2X) was secured to the midbody flank of each chameleon using Histoacryl^®^ tissue glue (Fig. 2). The transmitter was secured dorsally, with the whip antenna trailing posteriorly to minimize interference with the animals' movement. In all cases, the mass of the transmitter did not exceed 5% of an individual's body mass. After being fitted with a transmitter, chameleons were individually housed in Exo Terra® QuickRelease Full Screen Terraria (∼30 × 30 × 100 cm) supplied with ample vegetation for the remainder of the day to ensure that the transmitter was secure and that the animal did not show any negative behaviors associated with the transmitter (e.g., scratching at it, or otherwise attempting to remove it). All radio-telemetered individuals were released that evening (<24 h after initial capture) on the branch where they were originally captured.

### Radio-tracking and behavioral observations

Each evening during a 10-day tracking period, we located the position of the radio-telemetered chameleons through triangulation using the Telonics TR-8 handheld radio receiver and RA-23K VHF antenna (Telonics Inc.). In addition, we also searched the study area for chameleons that were not radio-telemetered. These individuals were captured, measured, and marked with an ID on site and coordinates were recorded. Thereafter, they were released on the same branch. We did this for two reasons: 1) during observations, it allowed us to collect data on intraspecific interactions between focal individuals and any other nearby individuals that were only marked with an ID and 2) to collect observational data on additional chameleons that were not radio-telemetered (i.e., this increased our sample of chameleons for observations). For additional chameleons that were encountered each evening that had already been marked, only their position on the vegetation and coordinates were recorded.

Each morning, prior to the commencement of focal observations, we located as many marked individuals as possible either visually or by radio-telemetry. Focal observations began soon after sunrise, at ∼07h15 during the austral summer season and 08h00 during austral autumn. Observations for the day concluded between 17h00 and 17h30 when natural light began to diminish. In some cases, telemetered animals were the focus of observations for the morning period, and in other cases, the additional ink-marked (but not telemetered) individuals were observed. Prior to the focal observation period for each individual, we recorded the air temperature (T_a_) and noted the chameleons' position in the vegetation. Thereafter, between two and four observers were each stationed ∼5–10 m from each focal chameleon. Each observer viewed a focal chameleon's movements using binoculars (focal animal sampling). Given that Brümmer Park is a busy thoroughfare for walkers, runners, and cyclists, chameleons appeared to be habituated to human presence and did not react to humans or dogs passing in close proximity. Thus, our presence did not appear to disturb the chameleons.

Each observation period lasted 60–180 minutes and behaviors relating to movement within different categories were recorded: 1) thermoregulation: basking, movements into and out of sunlight, perching position, 2) foraging: number of feeding events and whether the feeding events were made whilst the chameleon was stationary or in motion, 3) interactions: intraspecific interactions, and 4) generalized walking: walking around with no obvious motivation. In addition, for chameleons that interacted with conspecifics, display behaviors (e.g., color changes and head bobbing) were also recorded. The start and end time of each observation period was recorded, as was the start and end time of each behavior. Following the conclusion of each focal observation period, the next chameleon was located (usually through telemetry) and another observation period was carried out. The use of radio-telemetry ensured that we were able to conduct observations on up to six chameleons per day, while observations on the individuals that were not radio-tracked were considered opportunistic but provided the advantage of increasing the sample of chameleons from the population. On occasion, some individuals were observed more than once during a single day. Each telemetered individual was recaptured at the end of 10 days in advance of the estimated 12-day battery life of the transmitters. Transmitters were removed with diluted acetone applied to the tissue glue. We successfully retrieved all radio-transmitters from each field season.

### Statistical analyses

All statistical analyses were conducted in *R* 4.4.3 (R Core Team 2024).

#### Behavioral analysis

The total time that chameleons spent moving and in stationary positions (perched in sunlight/exposed, perched in shade/foliage, perched in dappled light) per observation period was calculated by subtracting the start time from the end time of each behavior. Because multiple observation periods for each chameleon were conducted, the total time that each chameleon was observed for during the study period was calculated. Similarly, the total duration of each recorded behavior for each chameleon across all their respective observation periods was calculated. In addition, all stationary behaviors were grouped and the total time that chameleons spent being stationary, regardless of their position, during observation periods was calculated. Thereafter, the percentage of time spent moving (%TM), the percentage of time spent being stationary (grouped positions; %TS), and the percentage of time spent in each stationary position were calculated as a percentage of the total duration of the observation time of each chameleon. These data were partitioned into the different time periods (i.e., morning and afternoon). Importantly, for chameleons that were interacting with one another, there were instances in which the individuals did not show obvious walking and/or stationary behaviors. In these cases, only the sum of stationary and moving behaviors were used to estimate the percentage of time spent at each class of behavior and not the duration of the trial.

Before testing for differences between %TM and %TS, we carried out exploratory analyses to test for differences between sexes using the Wilcoxon rank sum test (data did not meet the assumptions of normality). No significant difference between sex was detected (*P* = 0.99) and thus sex was excluded as a factor in subsequent analyses for %TM and %TS. We therefore tested for differences between %TM and %TS using a two-factor aligned rank transformed analysis of variance (ART ANOVA; Wobbrock et al. 2011) with behavior (moving and stationary) and time-period (morning and afternoon) included as fixed factors. A separate ART ANOVA was also carried out to test for differences in the percentage of time spent between the different classes of behaviors (i.e., moving, and all types of stationary behaviors) during the different time periods.

#### Foraging behavior (percentage of attacks and attack rates)

The total number of feeding events for each chameleon was calculated for each chameleon's entire observation period. Feeding events were grouped according to how the attack on prey was made (hereafter referred to as attack type); attack while stationary (AWS) and attack while moving (AWM). Thereafter, we calculated the percentage of attacks (PAS and PAM) for each individual and partitioned the data into the different time periods. We tested if sex should be included as a factor in the main analysis by first running an unpaired two-sample Wilcoxon rank sum test for percentage of attacks and because no significant difference was detected (*P* = 0.99), sex was excluded as a factor in the primary analysis. As our data did not meet the assumptions of normality, even after being arcsine-square root transformed, we used a two-factor ART ANOVA to compare the percentage of attacks between attack types and time periods.

We also assessed the rate of attacks on prey to validate our findings of the percentage of attacks. To do this, we calculated the attack rate of each individual over their entire observation period for AWS and AWM and scaled the attack rates such that they were as a measure of the number of attacks on prey per hour. The data were partitioned into the time periods for analysis. Again, we first tested for differences between sex using an unpaired two-sample Wilcoxon rank sum test. No significant difference in attack rate was detected between sex (*P* = 0.87) and thus sex was excluded as a factor in the subsequent analysis. Our measures of attack rate failed to meet the assumptions of normality even after being log-transformed. Thus, the two-factor ART ANOVA was carried out to compare differences in the attack rate between the attack type and time periods.

## Results

The behaviors of 38 individual *Bradypodion pumilum* (♀ = 17, ♂ = 19, unknown = 2) were observed and recorded for a total observation time of ∼171 h (Table 1). Although the sex ratio was marginally skewed in favor of male chameleons, the total observation time of female chameleons was more than that for males (Table 1). We observed two individuals that were encountered opportunistically but were not captured due to losing sight of them and thus their sex was unknown. A total of 4 h of observations were made on unknown chameleons.

**Table 1:** The total observation hours of female and male individuals of bradypodion pumilum.

Sex	Number of chameleons	Total observation time (h)
♀	17	102.0
♂	19	65.1
Unknown	2	4.0
**Total**	38	171.1

♀=Female; ♂ = Male.

### Movement and stationary behavior

**Figure 3:**
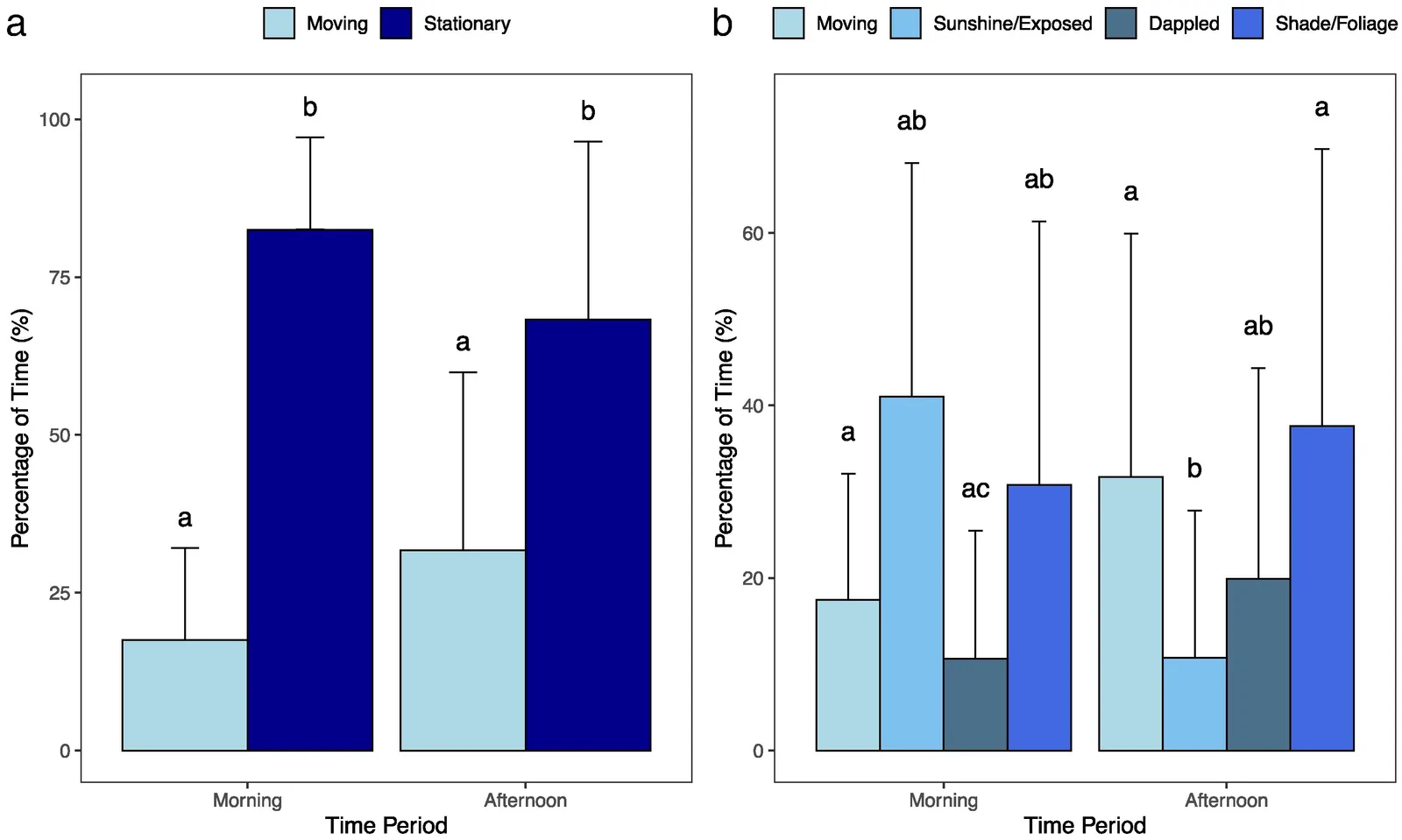
Bar graphs showing the percentage of time that individuals of *Bradypodion pumilum* (*n* = 38) spent within different behavioral classes, during the mornings and afternoons. (a) Represents the percentage of time spent moving (%TM) and being stationary (%TS), while (b) shows %TM and percentage of time spent being stationary in different thermal environments. Bars represent the means (+1 SD). Letters above each bar represent significant differences between behaviors within each time period. Bars that share any letters represent behaviors that are not statistically different from one another.

Over the entire day, chameleons spent a significantly greater (ART ANOVA: *F*_(1,104)_ = 126.42, *P* ≤ 0.001) percentage of time stationary (%TS; x̄ ± SD = 76.19 ± 22.65%) than they did moving (%TM; x̄ ± SD = 23.81 ± 22.65%; Fig. 3a). There was also a significant interaction for the time-period (i.e., morning or afternoon) and %TS and %TM (ART ANOVA: *F*_[1,104]_ = 10.29, *P* ≤ 0.05), but time-period alone did not yield a significant result (*P* = 0.99). Chameleons had a higher %TS (i.e., in perched positions) than %TM in both the mornings and afternoons, but the proportions between time periods for %TM and %TS were similar (Fig. 3a).

The ART ANOVA revealed a significant difference in the percentage of time spent in each behavioral class (i.e., moving and all stationary behaviors) by the chameleons (*F*_[3,208]_ = 6.45, *P* ≤ 0.001), a significant interaction of behaviors and time-period (*F*_[3,208]_ = 10.01, *P ≤* 0.001), but no significant difference in time-period alone (*P* = 0.39). Chameleons spent more than 80% of the time in stationary positions in the mornings, with the most time (x̄ ± SD = 41.04 ± 27.09%) perched in exposed areas in direct sunlight (Fig. 3b). The remainder of time in the mornings was spent being perched in shaded/vegetated (x̄ ± SD = 30.81 ± 30.55%) areas or in areas with dappled light (x̄ ± SD = 10.67 ± 14.83%). In contrast to these stationary positions during the morning, chameleons spent only a small percentage of time moving (x̄ ± SD = 17.48 ± 14.59%). Compared to the mornings, chameleons spent the least percentage of time in exposed areas (x̄ ± SD = 10.75 ± 17.07%; *P ≤* 0.001) during the afternoon (Fig. 3b). While the percentage of time spent in shaded/vegetated and dappled light positions increased during the afternoons, pairwise comparisons against the morning positions did not reveal significant differences. In addition, although the %TM in the afternoons (x̄ ± SD = 31.73 ± 28.22%) was almost double that during the mornings, the difference was not significant (*P* = 0.77). The %TM in the afternoons was low compared to the ∼70% of time that was spent in the stationary positions combined (Fig. 3).

### Foraging behavior

**Figure 4:**
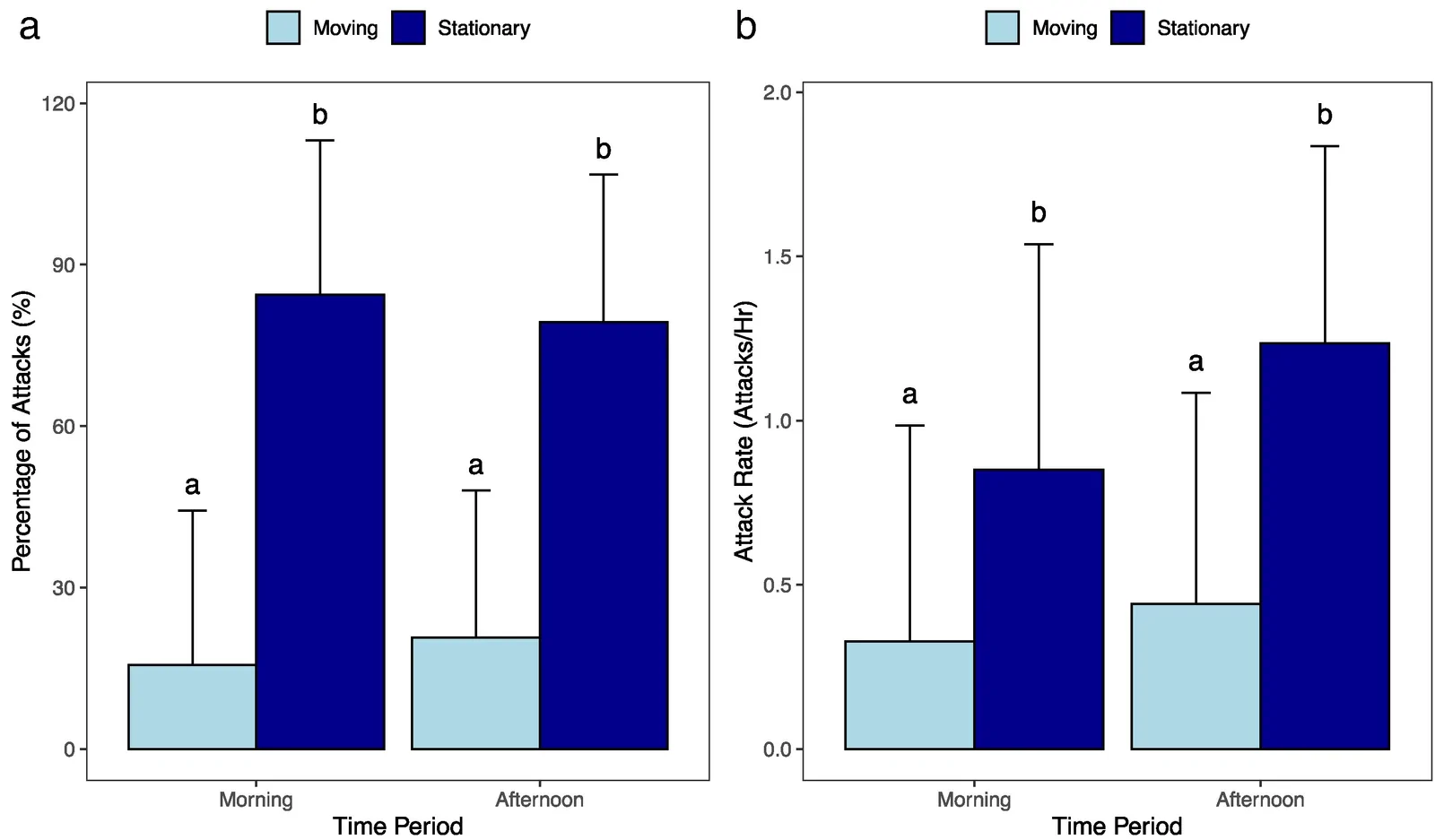
Bar graphs showing attack-based indices, a proxy for feeding events, for *Bradypodion pumilum* (*n* = 26) during the mornings and afternoons. (a) Represents the mean (+ 1SD) percentage of attacks made on prey while individuals were moving (PAM) and while they were stationary (PAS). (b) Shows the mean (+1SD) rate of attacks on prey while chameleons were moving and while they were stationary. Letters above each bar represent significant differences between behaviors within each time period. Bars that share any letters represent behaviors that are not statistically different from one another.

We recorded a total of 110 feeding events from 26 chameleons (♀ = 13, ♂ = 12, Unknown = 1) over the study period. Chameleons had a significantly higher percentage of attacks while stationary (PAS) compared when they were moving (PAM; ART ANOVA: *F*_[1,66]_ = 84.70, *P ≤* 0.001; Fig. 4a). Over the entire day, 81.94 ± 27.77% (x̄ ± SD) of attacks were made while stationary. In addition, the PAS did not differ significantly between the morning and afternoons (*P* = 0.97), nor did the percentage of attacks made while moving (PAM; *P* = 0.97). Even though the PAM increased marginally (∼5% increase) during the afternoon, the PAM was still considerably lower than the PAS which was nearly 80% during the afternoons (Fig. 4a).

In general, the mean rate of attack on prey while chameleons were stationary (x̄ ± SD = 1.04 ± 0.66 Attacks/h) was more than double and significantly higher (ART ANOVA; *F*_(1,66)_ = 29.01, *P ≤* 0.001) than their rate of attack on prey while they were moving (x̄ ± SD = 0.38 ± 0.64 Attacks/h; Fig. 4b). Attack rates were also significantly higher (increase of > 0.20 Attacks/h) during the afternoon compared to the morning (ART ANOVA; *F*_(1,66)_ = 8.16, *P ≤* 0.05), but the highest attack rate in both the morning and afternoon periods was while chameleons were stationary (Fig. 4b). There was also no apparent difference in attack rate between the morning and afternoon periods, regardless of how the attack was made.

### Observational interactions

**Figure 5:**
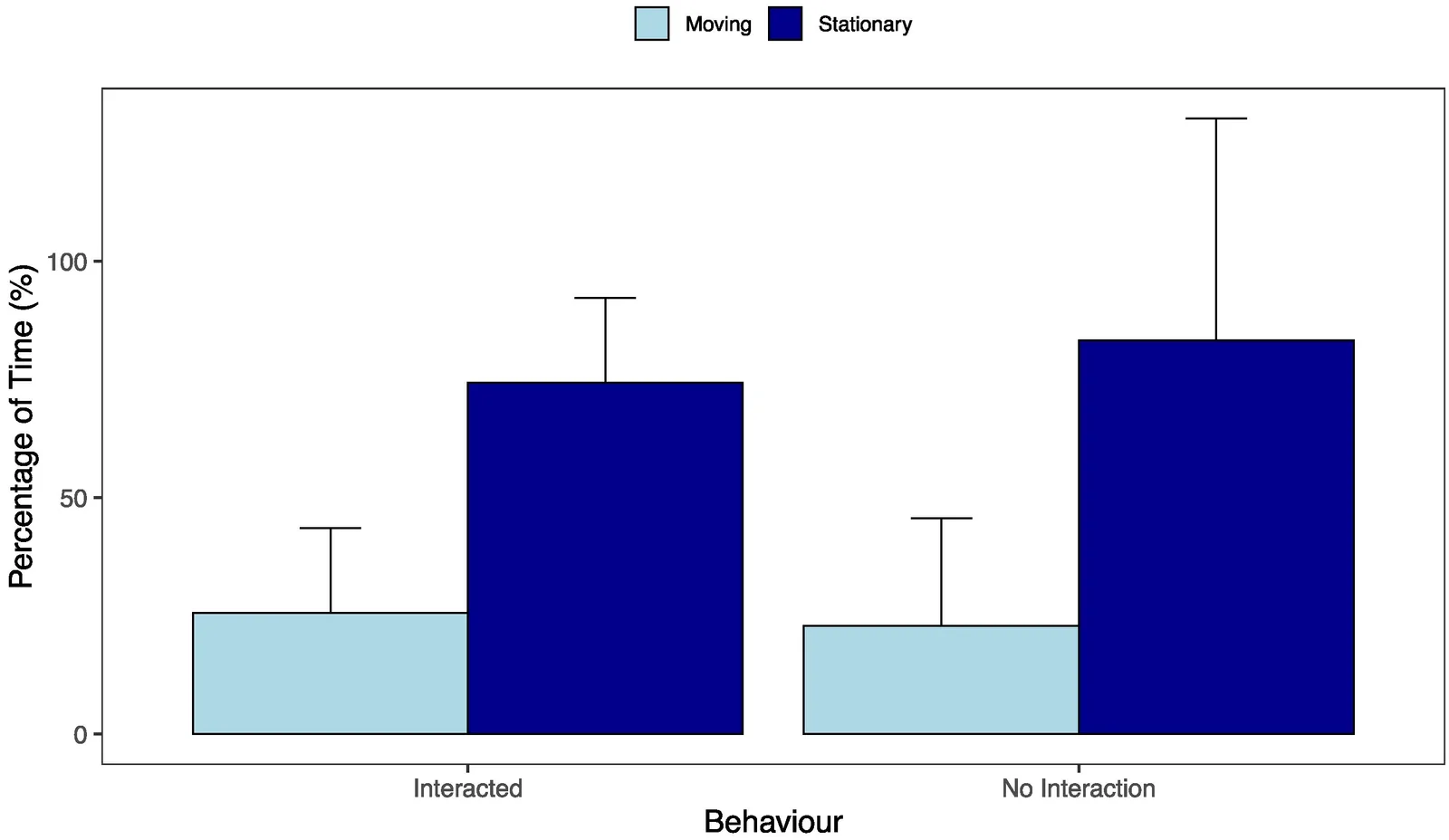
Bar graph showing the mean (+1SD) percentage of time that interacting (*n* = 11) and non-interacting (*n* = 33) individuals of *Bradypodion pumilum* spent moving (%TM) and being stationary (%TS) over the whole day. Given the large difference in the sample size between interacting and non-interacting chameleons, a formal statistical test was not performed.

A total of 36 intraspecific interactions were recorded between 11 individuals during the study. The total number of interactions were similar in the morning and afternoon periods of the day with 19 and 17 interactions, respectively. In addition, there were no obvious differences in %TM and %TS between interacting and non-interacting chameleons (Fig. 5). However, given the small sample size (*n* = 11) of interacting chameleons compared to the number of individuals that had no interactions during an observation period (*n* = 33), statistical tests were not carried out, making these observations qualitative. In one instance, a male appeared to be guarding a female as he remained ∼5–10 cm above the female on a separate branch, positioning himself to mirror her movements for the total duration of an observation period. The majority of the remaining observations recorded related to interactions between female and male chameleons. Most often observed was that upon encountering a female, males would turn bright green in color and would perform lateral head bobbing displays. By contrast, females were usually aggressive toward males; they typically turned dark, would hiss at the male, threaten the male by mouth-gaping, and occasionally would chase the male. We recorded a single instance where a female forced an approaching male out of the tree in which both were perched, with the male falling to the ground. The male immediately climbed back up to the location in the tree, repositioning himself near to the female.

## Discussion

Contrary to predictions based on a cruise foraging model, we found that chameleons spent the majority of time stationary throughout the day, foraging opportunistically. During the mornings, they spent most of the time perched in direct sunlight, presumably to raise T_b_. While the percentage of time spent moving (%TM) increased during the afternoons, chameleons were still stationary most of that time, perched in shade, or in direct or dappled sunlight. Movements usually consisted of shuttling between these thermal microhabitats, suggesting that movements were motivated by thermoregulatory needs. In addition, the numerous interactions that we observed throughout the study period suggest that chameleons can be highly motivated to move to interact with conspecifics. Despite the higher %TM during the afternoons, examination of feeding events revealed that most feeding events, and the highest rate of attacks made on prey occurred while individuals were stationary. Although chameleons occasionally foraged while moving (AWM), the rates and percentages of AWM were low, suggesting that these foraging events while individuals were moving were opportunistic. Thus, our results suggest that *B. pumilum* is motivated to move for a variety of reasons, such as thermoregulation or intraspecific interactions, and that their foraging behavior is more consistent with that of an ambush forager. The use of attack-based metrics provides a new perspective on chameleon foraging mode which is in stark contrast to previous studies that suggested chameleons are cruise foragers (Butler 2005; Hagey et al. 2010).

Attack-based indices have rarely been used to assess foraging mode but should be more informative than movement-based assessments. This is because the metrics are directly related to foraging activity (Cooper et al. 1999; Williams and McBrayer 2011). Our comparison of the methods for *Bradypodion* support an incongruity between approaches, with diametrical outcomes and interpretations. We recorded a high PAS (>80%) and a low PAM throughout the day, pointing to most feeding events being made while chameleons were stationary. While Butler (2005) interpreted their results in support of *Bradypodion* employing cruise foraging, this was based primarily on differences amongst movement-based metrics (%TM and MPM). When considering their data on feeding events in terms of percentages, the PAS translates to ca. 73%. Thus, in both cases, the high measures of PAS showed that most feeding events were made while chameleons are stationary, which is inconsistent with the findings that chameleons are cruise foragers. In addition, although little information on the PAS for ambush foragers exists within the literature, our measure of PAS for *B. pumilum* was similar to that of another ambush foraging lizard, *Scleroporus woodi* (Williams and McBrayer 2011). Moreover, another study applied a threshold of PAM ≥ 50% to assign lizards as active foragers (Cooper et al. 2001). Following this approach, our measure for PAM (< 20%) would classify *B. pumilum* as an ambush forager. This appears reasonable considering that the metrics of PAM and PAS are proxies for the number of attacks on prey and the method used to capture the prey.

Attack rate is another important attack-based index but has infrequently been applied to the assessment of foraging mode. Our comparison of the rate of AWS with the rate of AWM revealed a substantially greater attack rate by *B. pumilum* while stationary which offers strong support for defining the species as an ambush forager, especially considering that this pattern was consistent throughout the day. The fact that our measure for the rate of AWM was low supports the low PAM that we recorded, suggesting that chameleons were not necessarily engaging in cruise or active foraging. Thus, while PAM and PAS provide an indication on the number of prey items consumed while stationary and moving, attack rate addresses the frequency of attacks made on prey and the preferred mode of making those attacks.

The rate of attacks on prey varies considerably between species and there does not appear to be a clear difference in the rate of attacks on prey between active and ambush foraging lizards (Vitt et al. 1997; Williams and McBrayer 2011; McElroy et al. 2012). This is not surprising considering that attack rates are dependent on the biology of a species, how frequently individuals feed, how often prey is encountered, the abundance of prey and seasonality. Therefore, there are no obvious thresholds of attack rates that can be applied to categorize a species as an ambush or active forager. However, when assessing the rate of attacks on prey, differences between the rate of AWS and the rate of AWM may be more important as foraging mode preference can be inferred. Surprisingly, this distinction does not seem to be widely applied in the literature with most studies that measure attack rate do so without recording whether the attacks on prey are while individuals are moving or while they are stationary (e.g., Vitt et al. 1997; Nemes 2002; Williams and McBrayer 2011). Not recording the mode of making an attack when reporting on attack rates only results in a measure of the frequency of attacks on prey which may be meaningless when drawing conclusions about a species foraging behavior. Distinguishing between the mode of attack when assessing attack rate also infers preference of foraging behavior which can facilitate robust conclusions about a species foraging mode, especially if there has previously been some uncertainty arising from movement-based indices (e.g., Butler 2005).

Clearly, attack-based indices offer an informative assessment on foraging mode, but it must be noted that assessments on feeding behavior are difficult to achieve as attacks made on prey can be infrequent and require substantial observation time. This is likely the reason why most studies rely on movement-based metrics to assess foraging mode (Cooper et al. 1999). However, movement-based indices do provide important information about a species ecology but can be problematic when used in isolation to assess foraging behavior. This is because movements relating to thermoregulation, mate searching and predation avoidance generally make up parts of measurements of MPM and %TM which may result in erroneous interpretations of a species foraging mode. Our study, along with several others (Cooper et al. 1999, 2001; Williams and McBrayer 2011), has highlighted the value that attack-based indices (metrics that directly relate to foraging behavior) adds to foraging mode assessments. However, the most informative and realistic assessment of foraging mode is that which is multifaceted and includes movement- and attack-based indices, as well as accounts for the species life history.

The high proportion of time chameleons spent stationary in areas exposed to direct sunlight in the mornings fits their thermal biology as diurnal ectotherms. We suggest that they dedicate more time to thermoregulate in the mornings to increase T_b_ in order to reach selected or target temperature (Bennett 2004). A T_b_ at, or close to, target level is important so that certain behaviors (e.g., foraging, predator avoidance, mate searching, etc.) and physiological functions (e.g., digestion, reproduction, etc.) can occur efficiently within the range of thermal optima (Seebacher 2005). In addition, while chameleons had a high %TS in the mornings, the PAS and rate of AWS were also high suggesting that microhabitats chosen to thermoregulate (either in sunshine or shaded patches) also offered ideal ambush sites. Although movement did increase after midday, the combined proportion of time at the different stationary positions (perched in sunshine, perched in shade, perched in dappled light) was still proportionally greater. This indicates that movements were not driven solely by the need to forage, but that shuttling between different thermal microenvironments to maintain T_target_ may also explain the increased movements observed during the afternoons.

An unexpected finding was that both our measures and those of Butler (2005) for %TM (ca. 20%) were greater than the proposed threshold of 15% for active foragers (Huey and Pianka 1981; Cooper and Whiting 1999). This would classify *B. pumilum* as an active forager. However, the number of MPM recorded by Butler (2005) was fewer than 0.5 MPM, much lower than the proposed threshold of MPM *≤* 1 for an ambush forager (McLaughlin 1989). By this metric, *B. pumilum* would be considered an ambush forager. This dichotomy appeared to be the motivation to classify *B. pumilum* under the category of cruise forager (Butler 2005), neither fully active nor fully ambush. Based on these same metrics, *Trioceros jacksonii* was also concluded to be a cruise forager (Hagey et al. 2010). However, chameleons have slow, extremely drawn-out movements that can take minutes to complete. This makes it difficult to partition their movements into a dichotomy of moving or stationary upon which the MPM and %TM metrics are based. Given this, the MPM are expected to be low. In addition, an elevated %TM could be attributed to their style of movement of which may be mistaken for individuals moving into areas to thermoregulate, to find alternative sites to station themselves in ambush, to avoid predation, or to interact with conspecifics. Moreover, it must be noted that the thresholds proposed for MPM and %TM are arbitrary. Thus, because movement can and does relate to non-foraging activities, MPM and %TM may not always be the most appropriate metrics to assess foraging mode, particularly if used in isolation without other metrics.

Being classified as a cruise forager (Butler 2005; Hagey et al. 2010) does not easily correspond to aspects of chameleon morphology, physiology, or behavior, most of which are typical of an ambush forager. Chameleons have excellent camouflage (Stuart-Fox et al. 2008; Stuart-Fox and Moussalli 2009), are slow-moving (Hagey et al. 2010; Herrel et al. 2013), and use their projectile tongue to catch their prey (Anderson 2016). All these characteristics are consistent with an ambush foraging mode rather than cruise foraging where targeted movement toward prey would be compulsory. Furthermore, the diet of *B. pumilum* (from the same population as our study) consists primarily of active prey as opposed to sedentary prey (Measey et al. 2011). This trait is consistent with ambush foraging, as active prey spend most of their time moving and could wander into striking range of a chameleon hiding in ambush. In contrast, a diet of sedentary prey would mean that prey items would need to be actively pursued. This suite of life-history traits with our findings of high %TS, PAS, and rate of AWS for *B. pumilum* support a paradigm shift, that chameleons rely on an ambush foraging strategy and should not be regarded as cruise foragers.

The intraspecific interactions that we observed and recorded suggest that *B. pumilum* may also be motivated to move to interact with or avoid conspecifics. Most of these interactions were between sexes and were presumably related to attempts from males to draw the attention of females for the purposes of mating. Given that spermatogenesis and vitellogenesis in *Bradypodion* is aseasonal, but with peaks during the austral autumn and spring (Jackson 2007), it might be expected that behaviors associated with mating are exhibited year-round but could be more frequent during warmer months. This might explain why we recorded more interactions during our austral autumn field season compared to that during the summer. Nevertheless, interactions were recorded during both field seasons and were in line with chameleon life-history traits. Although the number of interactions that we recorded are in contrast to Butler (2005) who did not report any intraspecific interactions (during November, end of austral spring) the difference in observations may be due to the relatively short period of time (20–30 min compared to our 60–180 min sessions) during which individuals were observed and collectively the short total study period (9.5 h compared to our ∼171 h) in the Butler (2005) study. Regardless, our observations suggest that movement in *B. pumilum* may also be motivated by interactions with conspecifics, presumably for reproductive reasons.

Previous studies on foraging mode in chameleons (e.g., Butler 2005; Hagey et al. 2010) placed emphasis on movement-based indices for assessing foraging mode; MPM and %TM. Given our findings that chameleons are motivated to move for several reasons, movement-based indices, when used in isolation, are inadequate for classifying foraging mode. Our case study addressed the limitations by categorizing the time that individuals dedicate to thermally motivated behaviors and movement, acknowledged intraspecific interactions and placed strong emphasis on attack-based indices. In addition, most studies that use attack-based indices seldom include measures of the rate of attacks on prey, and those that do generally do not assess the mode of attack. We argue that attack rate is an important metric that can validate findings arising from percentage of attacks. After considering the species life history, the high %TS, and high PAS and rate of AWS, it can be concluded that cruise foraging does not easily explain foraging mode for *B. pumilum* whereas an ambush foraging strategy appears to be more applicable. More generally, we propose that an overall paradigm shift regarding foraging mode in chameleons is needed, and that they be considered as ambush foragers rather than cruise foragers. Our study suggests that contextualizing foraging strategies within a full gamut of behaviors assessed by multiple indices should provide a more meaningful classification of foraging mode across the board.

## Data Availability

The data used in this study are available at: https://doi.org/10.6084/m9.figshare.30208804.v1.
